# Placement of Posterior Composite Restorations: A Cross-Sectional Study of Dental Practitioners in Al-Kharj, Saudi Arabia

**DOI:** 10.3390/ijerph182312408

**Published:** 2021-11-25

**Authors:** Mohamed M. Awad, Mansour Alradan, Nawaf Alshalan, Ali Alqahtani, Feras Alhalabi, Mohammed Ali Salem, Ahmed Rabah, Ali Alrahlah

**Affiliations:** 1 Department of Conservative Dental Sciences, College of Dentistry, Prince Sattam Bin Abdulaziz University, Al-Kharj 11942, Saudi Arabia; f.alhalabi@psau.edu.sa (F.A.); m.abuelqomsan@psau.edu.sa (M.A.S.); 2 College of Dentistry, Prince Sattam Bin Abdulaziz University, Al-Kharj 11942, Saudi Arabia; 437050135@std.psau.edu.sa (M.A.); 437050536@std.psau.edu.sa (N.A.); 437050749@std.psau.edu.sa (A.A.); 3 Department of Prosthetic Dental Sciences, College of Dentistry, Prince Sattam Bin Abdulaziz University, Al-Kharj 11942, Saudi Arabia; a.rabah@psau.edu.sa; 4 Department of Restorative Dental Science, College of Dentistry, King Saud University, Riyadh 11545, Saudi Arabia; 5 Engineer Abdullah Bugshan Research Chair for Dental and Oral Rehabilitation, College of Dentistry, King Saud University, Riyadh 11545, Saudi Arabia

**Keywords:** posterior restoration, composite, dental practitioners

## Abstract

Dental practitioner-related factors can affect the quality of composite restorations. This study aimed to investigate the clinical techniques used by dental practitioners (DPs) while placing direct posterior composite restorations. Methods: A questionnaire survey that sought information related to the placement of posterior composite restorations was delivered to 161 DPs working in the Al-Kharj area, Saudi Arabia. The collected data were statistically analyzed using Pearson’s Chi-square test and Fisher’s exact test considering the DP’s working sector and the answered questions. Results: A total of 123 DPs completed the survey (76.4% response rate). There was a statistically significant difference between DPs working in the private sector and those working in the governmental sector in 7 out of 17 questionnaire items namely: preparing a minimum depth of 2 mm, (*p* = 0.001); mechanical means of retention, (*p* = 0.003); operative field isolation, (*p* = 0.004); adhesive strategy, (*p* < 0.001); light-curing unit used, (*p* = 0.013); the use of radiometer, (*p* = 0.023), and dental matrix selection, (*p* < 0.001). Conclusion: The clinical techniques applied by DPs working in the private sector in Al-Kharj, Saudi Arabia when placing posterior composite restorations, including the specifications of cavity preparation, operative field isolation, and selection of the dental matrix system, may be substandard compared to those applied by DPs working in the governmental sector.

## 1. Introduction

Composite and dental amalgam have been used to restore class I and II cavities in the posterior teeth [1]. Resin-based composite (RBC) has gradually replaced amalgam over the past decade [2], while amalgam restorations have been questioned as they contain mercury [3,4]. Moreover, the shift toward minimally invasive management of carious lesions [5] and the improved physical and mechanical properties of composite restorations [6] resulted in the increased popularity of RBCs as posterior restorative materials. Each tooth undergoing an operative intervention is placed on a downward restorative spiral. The use of composites, in preference to amalgam, might help slow down this spiraling descent [7]. RBC has suitable composition and properties as the “material of choice” for use in direct posterior restorations [8]. The recognized advantages of composites over amalgam include avoiding sacrificing healthy tooth tissue to create mechanical undercuts [6] and increased fracture resistance of the restored tooth unit as teeth restored with amalgam are more susceptible to fractures [9,10]. Increased use of RBC has been noticed in countries such as Norway, Denmark, Finland, Germany, Italy, the USA, and Japan [11,12,13,14,15]. Indeed, a retrospective study of composite use in general practice found their 5- and 10-year survival rates to be slightly higher than amalgam [16]. Based on the findings of a meta-analysis that evaluated the longevity of posterior composite restorations, satisfactory clinical outcomes were noted. Recurrent caries and restoration fractures were the most common causes of restorative failure [17]. The success of composite resin restorations depends on understanding the physical properties and management of the resin material [18] However, the success or failure of composite restorations is not only a matter of material; rather, it is a multi-factorial process in which patient-related and operator-related factors are combined with technical aspects [17,19]. The dentist’s knowledge might be a crucial factor affecting technique-sensitive restorative procedures such as direct posterior composite restorations, affecting their longevity [20].

The use of questionnaire responses to determine dentists’ attitudes and practice is common [21]. A previous questionnaire indicated that most dentists in Northern Saudi Arabia preferred not to use RBC in class II cavities [22]. That study suggested more professional training on posterior composite restorations. In addition, private dental practitioners (DPs) in Riyadh, Saudi Arabia tend to replace existing amalgam restorations with RBC restorations [23]. The use of RBC materials as the dominant choice among dentists in Kuwait reflects the trend worldwide [4]. A similar finding has recently been noticed in New Zealand [24]. A recent study conducted in the United Kingdom indicated that RBC is the most used material for direct restoration of the premolars, whereas amalgam was used in the molar teeth [25]. While previous similar studies provided only descriptive statistics, this study assessed the relationship between DPs’ working sector and the clinical technique used by the DPs when placing posterior composite restorations. Moreover, no such similar study had been performed in Al-Kharj, Saudi Arabia. Therefore, this study aimed to investigate the clinical techniques used by DPs in Al-Kharj while placing direct posterior composite restorations.

## 2. Materials and Methods

### 2.1. Ethical Approval

The Research Ethics Committee in Health and Science Disciplines, Prince Sattam Bin Abdulaziz University (PSAU), Al-Kharj, Saudi Arabia, approved this study (approval No REC-HSD-37-2021). The study was conducted following the STROBE (Strengthening the Reporting of Observational Studies in Epidemiology) guidelines [26].

### 2.2. Questionnaire Description

Based on a previous study [7], an electronic questionnaire was developed using Google Forms, and a pilot version was validated and tested for usability using concurrent think aloud and verbal-probing approaches [27] by five university teachers of restorative dentistry from PSAU, King Saud University, Saudi Arabia, and five dental practitioners working in Ministry of Health hospitals and the private sector in Al-Kharj, Saudi Arabia. Following this preliminary trial, modifications were made to ensure appropriate preparation and clinical relevance of the questionnaire sections and questions. Based on the feedback of the participants in the pilot study, pictures were added to provide more clarification of two questionnaire questions namely: “Which material do you often use in posterior large cavity (3 or more surfaces)?” and “Do you bevel the gingival margin of the cavity?”. The questionnaire is consisted of a total of 17 closed ended questions with binary or multiple-choice answers and sought information related to the placement of occlusal class I and II posterior direct composite restorations. The questionnaire sections were as follows: (1) The information of the dental practitioners participated in the study, (2) The selection of restorative material and placement of composite in special cases, (3) The use of composite in special cases, (4) The specifications of cavity preparation for posterior composite restorations, and (5) The restorative technique applied during the placement of posterior composite restorations. The questionnaire items are provided in the Supplementary Materials.

### 2.3. Sampling and Data Collection

Based on the statistical yearbook (2020), Ministry of Health, Saudi Arabia [28], the number of DPs in Al-Kharj was estimated to be 179. The sample size (*n* = 123) was calculated at a confidence interval of 95% and margin of error of 5%. The questionnaire was electronically delivered to 161 dental practitioners working in a total of a total of 35 hospitals and/or dental clinics of which 7 (20%) are governmental and 28 (80%) are private located in Al-Kharj, Saudi Arabia between March to June 2021. The data were anonymously collected and authors had no access to the participants’ information.

### 2.4. Statistical Analysis

The collected data were statistically analyzed considering two variables, (1) dental practitioners’ working sector and (2) the answered questions using either Pearson’s Chi-squared test with Yates’ continuity correction or Fisher’s exact test with a significance level of 0.05 (R software 4.1.1, R Foundation for Statistical Computing, Vienna, Austria).

## 3. Results

### 3.1. Response Rate

A total of 123 dental practitioners with a response rate of 76.4% participated in the questionnaire, of whom 84 (68.3%) have more than 5 years of clinical experience and 86 (70%) are working in the private sector. The information on the dental practitioners who participated in the study are detailed in Table 1.

### 3.2. Selection of the Restorative Material

**Figure 1:**
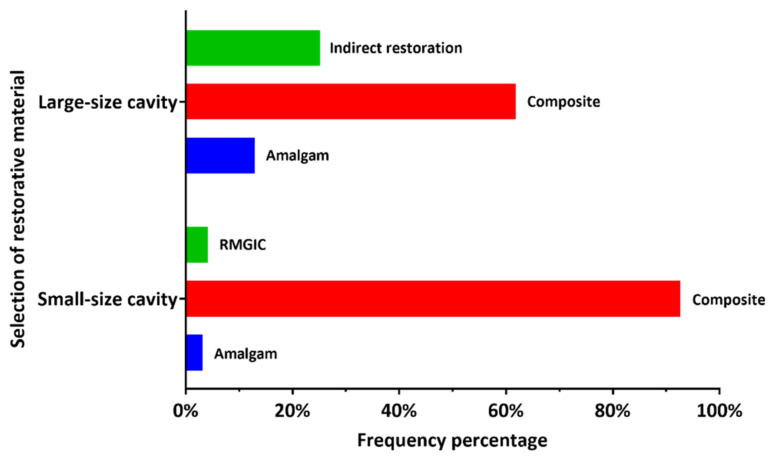
Dental practitioner-reported frequency of the selection of restorative material for small-size and large-size posterior cavity preparations (RMGIC: resin-modified glass ionomer cement).

The material of choice for posterior restorations according to the participant DP is graphically described in Figure 1. In addition, more detailed descriptive statistics and comparison between DPs working in the governmental and private sectors are provided in Table 2. Composite was the most used restorative material for direct posterior restorations in small-size one- or two-surface cavities as well as the large-size cavities involving three or more tooth surfaces, as reported by 114 (92.6%) and 72 (58.6%) dental practitioners. There was no statistically significant difference between the dental practitioners working in the governmental and private sectors (*p* > 0.5).

### 3.3. Placement of Posterior Composite Restorations in Patients with Certain Clinical Conditions

**Figure 2:**
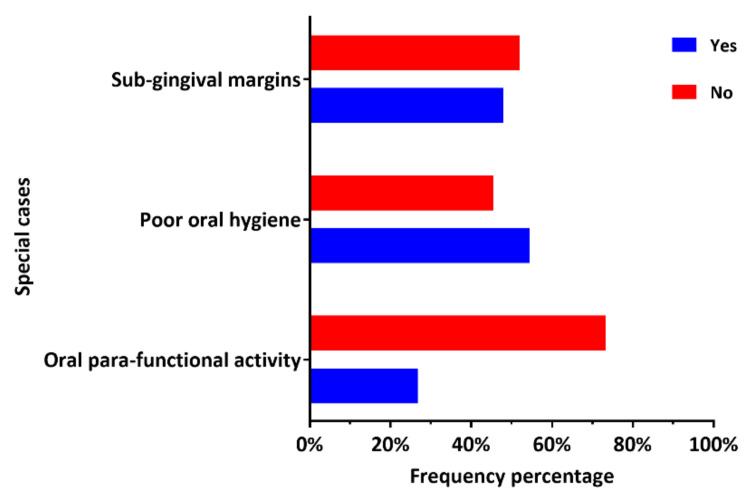
Dental practitioner-reported frequency of the placement of composite in special cases.

The use of composite in special cases as reported by the participants is graphically described in Figure 2. In addition, more detailed descriptive statistics and comparison between DPs working in the governmental and private sectors are provided in Table 2. The placement of posterior composite restorations in patients with occlusal parafunctional activity, with poor oral hygiene, and in cavities with subgingival margins was reported by 33 (26.8%), 67 (54.5%), and 59 (48%) dental practitioners, respectively. There was no statistically significant difference between the dental practitioners’ working in the governmental and private sectors (*p* > 0.5).

### 3.4. Specifications of the Cavity Preparation

**Figure 3:**
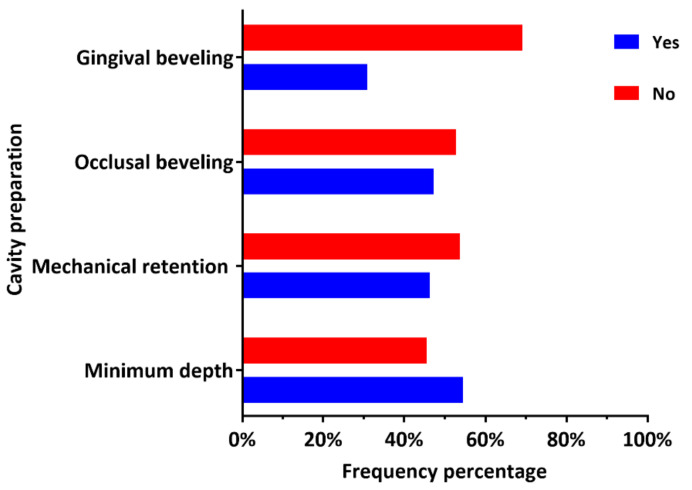
Dental practitioner-reported frequency of the specifications of the cavity preparation for posterior composite restorations.

The specifications of the cavity preparation for posterior composite restorations are graphically described in Figure 3. In addition, more detailed descriptive statistics and comparison between DP working in the governmental and private sectors are provided in Table 3. A total of 67 (54.5%) and 57 (46.3%) dental practitioners reported preparing a minimum depth of 2 mm and mechanical means of retention (undercuts) for posterior composite restorations, respectively. Beveling of the occlusal and gingival cavity margins was reported by 58 (47.2%) and 38 (30.9%) practitioners, respectively. There was a statistically significant difference (*p* < 0.05) between the dental practitioners’ working in the governmental and private sectors with regard to two specifications of cavity preparation for posterior composite restorations, namely the preparation of a minimum 2 mm pulpal depth (*p* = 0.001) and mechanical means of retention (undercuts) (*p* = 0.003).

### 3.5. Restorative Technique

**Figure 4:**
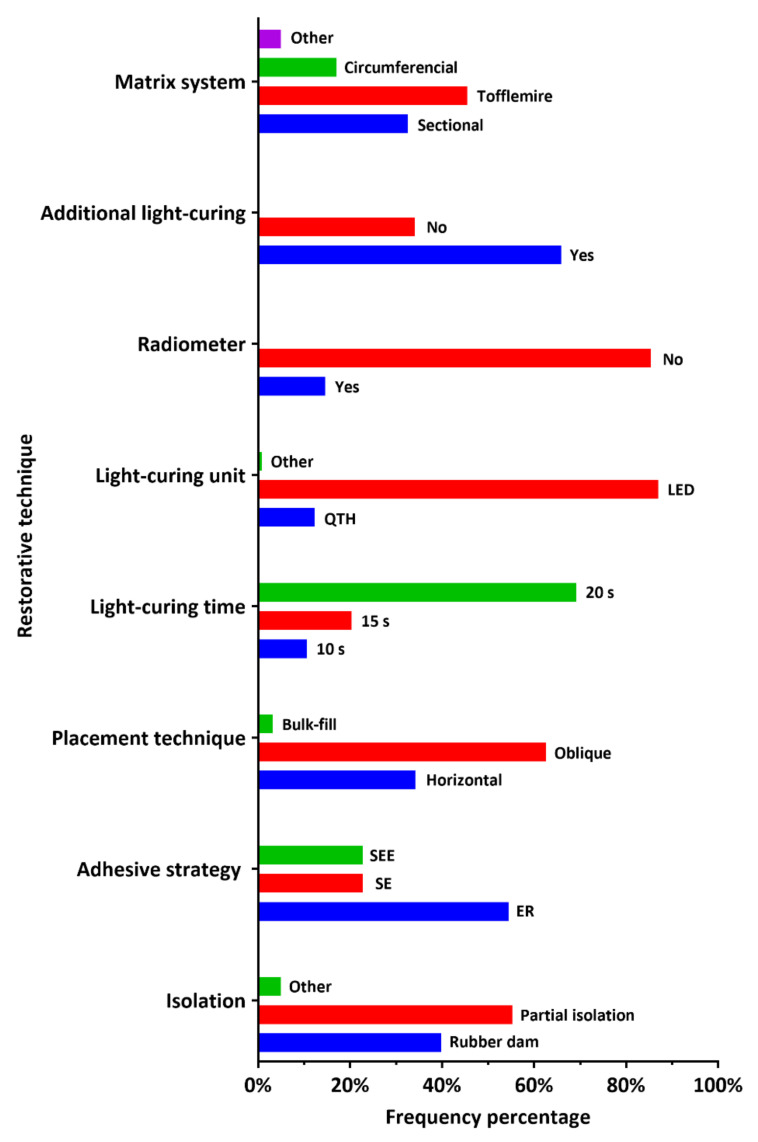
Dental practitioner-reported frequency of the restorative technique applied during the placement of posterior composite restorations (ER: etch-and-rinse, SE: self-etching, SEE: selective enamel etching, QTH: quartz tungsten halogen, LED: light emitting diodes).

Steps of the restorative technique as reported by the participant DP are graphically described in Figure 4. In addition, more detailed descriptive statistics and comparison between DP working in the governmental and private sectors are provided in Table 4. A total of 49 (39.8%) dental practitioners reported using a rubber dam for operative field isolation. The etch-and-rinse adhesive strategy was the most reported by 67 (54.5%) of the dental practitioners. A total of 77 (62.6%) dental practitioners reported the use of the oblique layering technique during the insertion of posterior composite restorations. The light curing of 2 mm composite layer (increment) for 20 s was reported by 85 (69.1%) of the dental practitioners. The light emitting diodes (LED) light-curing units were the most used for this purpose, as reported by 107 (87%) dental practitioners. Surprisingly, only 18 (14.6%) dental practitioners reported regular monitoring of the light-curing units by a radiometer. Additional light-curing intervals after the removing the metal matrix band were reported by 81 (65.9%) of the dental practitioners. The use of a Tofflemire metal matrix system for restoring the proximal contact with posterior composite restorations was reported by 56 (45.5%) practitioners, while only 40 (32.5%) and 21 (17.1%) reported the use of a sectional or circumferential matrix system, respectively, for the same purpose. There was a statistically significant difference between the dental practitioners’ working in the governmental and private sectors with regard to a total of five steps of the restorative technique applied during the placement of posterior composite restorations, namely the method of operative field isolation (*p* = 0.004), the adhesive strategy used (*p* < 0.001), the light-curing unit type (*p* = 0.013), the regular use of radiometer to monitor the output of the light-curing unit (*p* = 0.023), and the selection of the matrix system for restoring the proximal contact with composite restoration (*p* < 0.001).

## 4. Discussion

The increased number of failed posterior composite restorations in patients screened at the clinics of the College of Dentistry, PSAU motivated the authors to conduct this cross-sectional study to investigate the clinical techniques used by the DP in Al-Kharj while placing direct posterior composite restorations. The comparison between DP working the governmental and those working the private sectors is relevant because of the fact that the incidence of dental malpractice may be higher in private clinics compared to governmental hospitals or centers [29]. The questionnaire used in this study was formulated, validated, and delivered electronically to the participants and was collected by the authors, resulting in a response rate of 76.39%, higher than the acceptable response rate suggested by Tan and Burke [21]. Most participants worked in the private sector (69.9%) and reported having over 5 years of clinical experience (68.3%).

Composite was the most popular choice among the study participants for restoring posterior cavities, regardless of the cavity size or extension. This follows the Academy of Operative Dentistry—European Section guidelines on posterior composite restorations that consider composite the most preferred restorative material to restore small and large cavities in the posterior teeth [8]. This approach is supported by the well-known advantages of composite restorations, including conservative cavity preparation and ease of repair compared to amalgam restorations, while meeting the increase in esthetic demands by the patients. Clinical studies have proven the excellent clinical performance and survival rate of large- and small-size posterior composite restorations [30]. However, the risk of failure of composite restorations increased with the restoration size [17] due to the masticatory forces and stresses applied to it [31]. This might explain why composite was selected for restoring one- or two-surface posterior cavities by 92.7% of the participants, while 58.5% selected it for restoring extensive posterior cavities with three or more surfaces. Amalgam was considered for restoring extensive cavities by 13% of the participants. A retrospective study performed in Finland indicated that composite and amalgam restorations might have similar clinical performance in 3-surface cavities [32]. The longevity of composite restorations could be affected by patient-related factors such as oral hygiene status [33] and parafunctional occlusal habits [34]. Such factors were associated with high failure rates of posterior composite restorations [17,34]. Dental practitioners seem concerned about the patients’ parafunctional habits as most (73.2%) denied the placement of posterior composite restorations in such cases. More than half (52%) of the participants preferred not to place posterior composite restorations in cavities with subgingival margins. It is well-known that the position of the cavity margins (subgingival) could complicate the clinical procedure in class II composite restorations due to the limited accessibility and absence of enamel in some cases [35,36]. However, clinical management of the interproximal papilla (gingiva) could facilitate field isolation, dental matrix placement, and composite insertion and decrease the chance for interproximal overhang formation [37]. Additionally, the results of a recent clinical and histological study indicated that composite restorations with subgingival margins were compatible with gingival health [38].

The cavity preparation could influence the quality of posterior composite restorations, although the effect of inappropriate cavity preparation would not be visible immediately after the restoration insertion [39]. Despite that, there is no consensus on teaching the principles of cavity preparation for posterior composite restorations in North America [40] or Saudi Arabia [41]. Beveling of occlusal cavity margins seems to be confusing to the dental practitioners in Al-Kharj area as almost half (47.2%) of them reported beveling the occlusal cavity margins during preparations for posterior composite restorations. Beveling of the occlusal margins should be contraindicated [8,42], as it might result in unnecessary loss of non-carious tooth structure and confusion during restoration finishing, repair, or replacement [6,8,43]. Approximately one-third (30.9%) of the participants reported beveling of gingival cavity margins, which might also lead to the loss of sound tooth structure [44] and affect the marginal integrity of composite restorations. It might also create composite flashes in the interproximal space, complicating the restoration finishing procedures due to limited accessibility [43]. Surprisingly, creating mechanical means for retention and a minimum depth of 2 mm of composite restoration were reported by many participants (46.3% and 54.5%, respectively), unnecessary for posterior composite restorations [42].

Isolation of the operative field with a rubber dam is recommended [45]. However, the placement of a rubber dam might be considered a time-consuming procedure for many dentists. Less than half of the participants (39.8%) reported using a rubber dam for operative field isolation. Clinical studies found no difference in the survival rates of posterior restorations where isolation was done with cotton rolls and aspiration or a rubber dam [46,47], However, applying a rubber dam before cavity preparation is highly recommended to enhance visibility and prevent contamination during the restorative procedures [39]. Composite restorations placed under rubber dam isolation showed significantly fewer defects that required replacement, enhancing their clinical longevity [45]. Moreover, during the COVID-19 pandemic, a rubber dam could protect the operating dentist [48].

Achieving durable bonding to tooth structures is indispensable to enduring posterior composite restorations. Three main adhesive strategies are applied in restorative dentistry. First, the etch-and-rinse strategy involves applying phosphoric acid etchant to demineralize the tooth structure before adhesive application [49]. Despite the adequate bond strength achieved with this strategy, matrix metalloproteinases could be activated during phosphoric acid etching or adhesive application [50]. Such enzymes could markedly deteriorate the resin-dentin bond strength [51]. Nevertheless, more than half of the participants (54.5%) reported this as the most often used strategy. Second, the self-etching strategy utilizes a primer or an adhesive with certain acidity to simultaneously demineralize and infiltrate the tooth structures instead of phosphoric acid etching [52]. However, this approach cannot achieve adequate bonding to the dental enamel. Third, the selective enamel etching strategy involves phosphoric acid etching of the enamel (cavity margins) followed by the application of a universal or multi-mode adhesive to the entire cavity (enamel and dentin) [53,54]. Selective enamel etching could improve the bond strength of universal adhesives to tooth structure [55].

The effectiveness of sectional matrix systems in generating a tight proximal contact in Class II composite restorations was proven [56,57,58]. However, this is not the most popular system in the UK [25]. Similarly, only 32.5% of the participant reported using a sectional matrix system, while more participants (45.5%) used the Tofflemire matrix system more often. The Tofflemire matrix system was associated with flaws in creating tight and properly contoured proximal contacts [59]. The more frequent use of a circumferential matrix to restore the proximal contact in class II composite restorations was reported by 17.1% of the participants. The incidence of food packing in the interproximal area was higher when the circumferential matrix was used [25], impairing the periodontal health and increasing the periodontal pocket depths [60].

Incremental placement of composite restoration is recommended to ensure adequate light-curing and overcome the polymerization stress effect [61]. Furthermore, the oblique layering technique achieves better bonding than either the horizontal increments or bulk placement [62,63]. Connecting the facial and lingual walls during the curing of composite increment or layer in horizontal incremental techniques might lead to a greater cuspal deflection [64]. Most participants (62.6%) reported using oblique layering most often, reflecting adequate knowledge of polymerization shrinkage stress.

Light curing units are susceptible to reductions in the quality and intensity of the light output, resulting in reduced curing potential and, in turn, compromised final restoration quality [8]. Most participants (87%) reported using light emitting diodes (LED) light-curing units, while only 12.2% reported using quartz tungsten halogen (QTH) light-curing units. A previous study assessed the light-curing units in hospitals of Riyadh, Saudi Arabia, showing that a higher percentage of the QTH units had suboptimal (reduced) light intensity [65]. Periodic monitoring of the light output intensity by dental radiometers was recommended to detect output changes [66,67]. Nevertheless, most participants (85.4%) reported that they do not regularly use dental radiometers. This finding agrees with a previous report from the Riyadh area, Saudi Arabia [68]. Another study [41] found that dental radiometer use was taught in just a few dental schools in Saudi Arabia. The use of metal matrix bands while restoring class II cavities might prevent the curing light from reaching certain areas of the composite restoration. For this reason, additional light-curing sessions from the buccal and lingual sides are recommended after removing the metal matrix bands [69,70]. Most participants (65.9%) reported performing such additional light-curing intervals. One of the limitations of this study is that it covered dental practitioners from just one Saudi governorate (Al-Kharj). Therefore, extrapolation of the findings to the entire country should be done with caution. Nevertheless, this study could serve as a starting point to identify gaps in knowledge among dental practitioners to formulate national (Saudi) guidelines or protocols on the placement of direct posterior composite restorations. Until then, specialized workshops should be provided by national scientific societies, such as the Saudi Society of Restorative Dentistry, in order to update DPs’ technical knowledge on composite restorations. In addition, such workshops could identify and provide clinical solutions to the dental malpractice related to the placement of composite restorations.

## 5. Conclusions

Most dental practitioners (DPs) who participated in the study reported the placement of posterior composite restorations more than other restorative materials regardless of the size of cavity preparation. The clinical techniques applied by the DPs working in the private sector in Al-Kharj, Saudi Arabia when placing posterior composite restorations, including the specifications of cavity preparation, operative field isolation, and selection of the dental matrix system may be substandard compared to those applied by the DPs working in the governmental sector. Clinical practice guidelines on placement posterior composite restorations should be set and implemented by the Saudi Commission for Health Specialties in both private and governmental sectors.

## Figures and Tables

**Table 1:** The information of the dental practitioners (DPs) participated in the study.

DPs’ Information	Total *n* = 123 (100%)	Governmental *n* = 37 (30%)	Private *n* = 86 (70%)
Professional registration			
General Practitioner (GP)	76 (61.8)	16 (13)	60 (48.8)
Resident	5 (4.1)	0	5 (4.1)
Registrar	10 (8.1)	7 (5.7)	3 (2.4)
Senior registrar	11 (9)	7 (5.7)	4 (3.3)
Consultant	21 (17.1)	7 (5.7)	14 (11.4)
Years of clinical experience			
0–2 years	12 (9.8)	5 (4.1)	7 (5.7)
2–5 years	27 (22)	8 (6.5)	19 (15.4)
More than 5 years	84 (68.3)	24 (19.5)	60 (48.8)

**Table 2:** Comparison between DPs working in the governmental and private sectors with regard to selection of restorative material and placement of composite in special cases.

Question	Governmental *n* = 37 (30%)	Private *n* = 86 (70%)	*p*-Value
Q1. Which material do often you use in posterior small cavity (1 or 2 surfaces)?			0.452
Amalgam	0	4 (3.3)	
Composite	35 (28.4)	79 (64.2)	
Resin modified glass ionomer	2 (1.7)	3 (2.4)	
Q2. Which material do you often use in posterior large cavity (3 or more surfaces)?			0.746
Amalgam	4 (3.2)	12 (9.8)	
Composite	22 (17.9)	54 (43.9)	
Other (Indirect restoration)	11 (9)	20 (16.2)	
Q3. Do you often place direct posterior composite restorations in patients with oral para-functional activity?			1.000
Yes	10 (8.1)	23 (18.7)	
No	27 (22)	63 (51.2)	
Q4. Do you often place direct posterior composite restorations in patients with poor oral hygiene?			0.066
Yes	15 (12.2)	52 (42.3)	
No	22 (17.9)	34 (27.6)	
Q5. Do you often place direct posterior composite restorations in posterior cavities with 1–2 mm Sub-gingival margins			0.201
Yes	14 (11.4)	45 (36.6)	
No	23 (18.7)	41 (33.3)	

**Table 3:** Comparison between DP working in the governmental and private sectors with regard to the specifications of cavity preparation for posterior composite restorations.

Question	Governmental *n* = 37 (30%)	Private *n* = 86 (70%)	*p*-Value
Q6. Do you prepare a minimum pulpal depth of 2 mm for occlusal cavities?			0.001 *
Yes	11 (9)	56 (45.5)	
No	26 (21.1)	30 (24.4)	
Q7. Do you prepare mechanical means of retention for composite restorations?			0.003 *
Yes	9 (7.4)	48 (38.9)	
No	28 (22.8)	38 (30.9)	
Q8. Do you bevel the occlusal margins of the cavity?			0.120
Yes	13 (10.6)	45 (36.6)	
No	24 (19.5)	41 (33.3)	
Q9. Do you bevel the gingival margin of the cavity?			0.212
Yes	8 (6.5)	30 (24.4)	
No	29 (23.6)	56 (45.5)	

*: statistically significant difference (Pearson’s Chi-squared test).

**Table 4:** Comparison between DPs working in the governmental and private sectors in regard to the restorative technique applied during the placement of posterior composite restorations.

Question	Governmental *n* = 37 (30%)	Private *n* = 86 (70%)	*p*-Value
Q10. How often do you achieve the operative field isolation?			0.004 ^#^
Rubber dam	23 (18.7)	26 (21.1)	
Cotton rolls and intraoral suction (Partial isolation)	13 (10.6)	55 (44.7)	
Other	1 (0.8)	5 (4.1)	
Q11. Which adhesive strategy do you use more often?			<0.001 ^#^
Etch-and-rinse (total etch)	30 (24.4)	37 (30.1)	
Self-etching (no acid etching)	2 (1.7)	26 (21.1)	
Selective enamel etching	5 (4.1)	23 (18.7)	
Q12. Which placement technique do you often apply for the placement of composite restorations?			0.472
Horizontal layering	12 (9.8)	30 (24.4)	
Oblique layering	25 (20.3)	52 (42.3)	
Bulk-fill	0	4 (3.2)	
Q13. Which light-curing unit do you often use to light-cure posterior restorations?			0.013 ^#^
Quartz tungsten halogen (QTH)	9 (7.3)	6 (4.9)	
Light emitting diodes (LED)	28 (22.8)	79 (64.2)	
Other	0	1 (0.8)	
Q14. Do you regularly monitor the output of light-curing unit with a radiometer?			0.023 *
Yes	10 (8.1)	8 (6.5)	
No	27 (22)	78 (63.4)	
Q15. How long do you light-cure composite increment of 2 mm thickness?			0.784
10s	4 (3.3)	9 (7.3)	
15s	7 (5.7)	18 (14.6)	
20s	26 (21.1)	59 (48)	
Q16. For class II composite restorations, after removal of the matrix band, do you often perform additional light-curing from the buccal and lingual directions?			0.087
Yes	29 (23.6)	52 (42.3)	
No	8 (6.5)	34 (27.6)	
Q17. Which matrix system do you often use to restore the proximal contact with composite restoration?			<0.001 ^#^
Sectional matrix	19 (15.4)	21 (17.1)	
Tofflemire matrix	6 (4.9)	50 (40.6)	
Circumferential matrix	10 (8.1)	11 (9)	
Other	2 (1.7)	4 (3.2)	

*: statistically significant difference (Pearson’s Chi-squared test); ^#^: statistically significant difference (Fisher’s exact test).

## Data Availability

The data presented in this study are available on request from the corresponding author.

## Supplementary Materials

The following are available online at https://www.mdpi.com/article/10.3390/ijerph182312408/s1, Questionnaire questions.

## References

[1] Burke F.J., Mackenzie L., Sands P. (2013). Dental materials—What goes where? Class I and II cavities. Dent. Update.

[2] Sunnegardh-Gronberg K., van Dijken J.W., Funegard U., Lindberg A., Nilsson M. (2009). Selection of dental materials and longevity of replaced restorations in Public Dental Health clinics in northern Sweden. J. Dent..

[3] Fuks A. (2001). The use of amalgam in pediatric dentistry. Pediatr. Dent..

[4] Khalaf M.E., Alomari Q.D., Omar R. (2014). Factors relating to usage patterns of amalgam and resin composite for posterior restorations—A prospective analysis. J. Dent..

[5] Ritter A.V. (2008). Posterior composites revisited. J. Esthet. Restor. Dent..

[6] Roeters J.J., Shortall A.C., Opdam N.J. (2005). Can a single composite resin serve all purposes?. Br. Dent. J..

[7] Gilmour A.S., Latif M., Addy L.D., Lynch C.D. (2009). Placement of posterior composite restorations in United Kingdom dental practices: Techniques, problems, and attitudes. Int. Dent. J..

[8] Lynch C.D., Opdam N.J., Hickel R., Brunton P.A., Gurgan S., Kakaboura A., Shearer A.C., Vanherle G., Wilson N.H., Academy of Operative Dentistry European Section (2014). Guidance on posterior resin composites: Academy of Operative Dentistry—European Section. J. Dent..

[9] Watts D.C., el Mowafy O.M., Grant A.A. (1987). Fracture resistance of lower molars with Class 1 composite and amalgam restorations. Dent. Mater..

[10] Lynch C.D., McConnell R.J. (2002). The cracked tooth syndrome. J. Can. Dent. Assoc..

[11] Pink F.E., Minden N.J., Simmonds S. (1994). Decisions of practitioners regarding placement of amalgam and composite restorations in general practice settings. Oper. Dent..

[12] Friedl K.H., Hiller K.A., Schmalz G. (1995). Placement and replacement of composite restorations in Germany. Oper. Dent..

[13] Mjor I.A., Dahl J.E., Moorhead J.E. (2000). Age of restorations at replacement in permanent teeth in general dental practice. Acta Odontol. Scand..

[14] Forss H., Widstrom E. (2004). Reasons for restorative therapy and the longevity of restorations in adults. Acta Odontol. Scand..

[15] Hayashi M., Seow L.L., Lynch C.D., Wilson N.H. (2009). Teaching of posterior composites in dental schools in Japan. J. Oral. Rehabil..

[16] Opdam N.J., Bronkhorst E.M., Roeters J.M., Loomans B.A. (2007). A retrospective clinical study on longevity of posterior composite and amalgam restorations. Dent. Mater..

[17] Opdam N.J., van de Sande F.H., Bronkhorst E., Cenci M.S., Bottenberg P., Pallesen U., Gaengler P., Lindberg A., Huysmans M.C., van Dijken J.W. (2014). Longevity of posterior composite restorations: A systematic review and meta-analysis. J. Dent. Res..

[18] Sabbagh J., McConnell R.J., McConnell M.C. (2017). Posterior composites: Update on cavities and filling techniques. J. Dent..

[19] Demarco F.F., Correa M.B., Cenci M.S., Moraes R.R., Opdam N.J. (2012). Longevity of posterior composite restorations: Not only a matter of materials. Dent. Mater..

[20] Lucarotti P.S., Holder R.L., Burke F.J. (2005). Outcome of direct restorations placed within the general dental services in England and Wales (Part 3): Variation by dentist factors. J. Dent..

[21] Burke F.J., McHugh S., Randall R.C., Meyers I.A., Pitt J., Hall A.C. (2004). Direct restorative materials use in Australia in 2002. Aust. Dent. J..

[22] Akbar I. (2015). Knowledge and attitudes of general dental practitioners towards posterior composite restorations in Northern Saudi Arabia. J. Clin. Diagn. Res..

[23] Alkhudhairy F. (2016). Attitudes of dentists and interns in Riyadh to the use of dental amalgam. BMC. Res. Notes.

[24] Broadbent J.M., Murray C.M., Schwass D.R., Brosnan M., Brunton P.A., Lyons K.S., Thomson W.M. (2020). The Dental Amalgam Phasedown in New Zealand: A 20-year Trend. Oper. Dent..

[25] Bailey O., Vernazza C.R., Stone S., Ternent L., Roche A.G., Lynch C. (2020). Amalgam Phase-Down Part 1: UK-Based Posterior Restorative Material and Technique Use. JDR Clin. Trans. Res..

[26] STROBE Statement Guidelines. https://www.strobe-statement.org/download/strobe-checklist-cohort-case-control-and-cross-sectional-studies-combined.

[27] Geisen E., Romano Bergstrom J., Geisen E., Romano Bergstrom J. (2017). Chapter 6—Think aloud and verbal-probing techniques. Usability Testing for Survey Research.

[28] The Statistical Yearbook (2020), Ministry of Health, Saudi Arabia. https://www.moh.gov.sa/en/Ministry/Statistics/book/Pages/default.aspx.

[29] Ozdemir M.H., Saracoglu A., Ozdemir A.U., Ergonen A.T. (2005). Dental malpractice cases in Turkey during 1991–2000. J. Clin. Forensic Med..

[30] Borgia E., Baron R., Borgia J.L. (2019). Quality and Survival of Direct Light-Activated Composite Resin Restorations in Posterior Teeth: A 5- to 20-Year Retrospective Longitudinal Study. J. Prosthodont..

[31] Bohaty B.S., Ye Q., Misra A., Sene F., Spencer P. (2013). Posterior composite restoration update: Focus on factors influencing form and function. Clin. Cosmet. Investig. Dent..

[32] Palotie U., Eronen A.K., Vehkalahti K., Vehkalahti M.M. (2017). Longevity of 2- and 3-surface restorations in posterior teeth of 25- to 30-year-olds attending Public Dental Service—A 13-year observation. J. Dent..

[33] Kopperud S.E., Tveit A.B., Gaarden T., Sandvik L., Espelid I. (2012). Longevity of posterior dental restorations and reasons for failure. Eur. J. Oral. Sci..

[34] Pallesen U., van Dijken J.W. (2015). A randomized controlled 27 years follow up of three resin composites in Class II restorations. J. Dent..

[35] Dablanca-Blanco A.B., Blanco-Carrión J., Martín-Biedma B., Varela-Patiño P., Bello-Castro A., Castelo-Baz P. (2017). Management of large class II lesions in molars: How to restore and when to perform surgical crown lengthening?. Restor. Dent. Endod..

[36] Veneziani M. (2010). Adhesive restorations in the posterior area with subgingival cervical margins: New classification and differentiated treatment approach. Eur. J. Esthet. Dent..

[37] Bailey O., O’Connor C. (2019). Papilla management in sub-gingival, interproximal, direct composite restoration: A key step to success. Br. Dent. J..

[38] Bertoldi C., Monari E., Cortellini P., Generali L., Lucchi A., Spinato S., Zaffe D. (2020). Clinical and histological reaction of periodontal tissues to subgingival resin composite restorations. Clin. Oral. Investing..

[39] Peumans M., Politano G., Van Meerbeek B. (2020). Effective Protocol for Daily High-quality Direct Posterior Composite Restorations. Cavity Preparation and Design. J. Adhes. Dent..

[40] Zabrovsky A., Neeman Levy T., Bar-On H., Beyth N., Ben-Gal G. (2019). Next generation of dentists moving to amalgam-free dentistry: Survey of posterior restorations teaching in North America. Eur. J. Dent. Educ..

[41] Awad M.M., Salem W.S., Almuhaizaa M., Aljeaidi Z. (2017). Contemporary teaching of direct posterior composite restorations in Saudi dental schools. Saudi J. Dent. Res..

[42] Lynch C.D., Shortall A.C., Stewardson D., Tomson P.L., Burke F.J. (2007). Teaching posterior composite resin restorations in the United Kingdom and Ireland: Consensus views of teachers. Br. Dent. J..

[43] Lynch C.D., Frazier K.B., McConnell R.J., Blum I.R., Wilson N.H. (2010). State-of-the-art techniques in operative dentistry: Contemporary teaching of posterior composites in UK and Irish dental schools. Br. Dent. J..

[44] Lynch C.D., O’Sullivan V.R., Dockery P., McGillycuddy C.T., Rees J.S., Sloan A.J. (2011). Hunter-Schreger Band patterns and their implications for clinical dentistry. J. Oral. Rehabil..

[45] Heintze S.D., Rousson V. (2012). Clinical effectiveness of direct class II restorations—A meta-analysis. J. Adhes. Dent..

[46] Cajazeira M.R., De Saboia T.M., Maia L.C. (2014). Influence of the operatory field isolation technique on tooth-colored direct dental restorations. Am. J. Dent..

[47] Raskin A., Setcos J.C., Vreven J., Wilson N.H. (2000). Influence of the isolation method on the 10-year clinical behaviour of posterior resin composite restorations. Clin. Oral. Investing..

[48] Frankenberger R., Van Meerbeek B. (2021). Editorial: Rubber-dam—A blessing not only in the Covid-19 era. J. Adhes. Dent..

[49] Van Meerbeek B., Yoshihara K., Van Landuyt K., Yoshida Y., Peumans M. (2020). From Buonocore’s Pioneering Acid-Etch Technique to Self-Adhering Restoratives. A Status Perspective of Rapidly Advancing Dental Adhesive Technology. J. Adhes. Dent..

[50] Mazzoni A., Nascimento F.D., Carrilho M., Tersariol I., Papa V., Tjäderhane L., Di Lenarda R., Tay F.R., Pashley D.H., Breschi L. (2012). MMP activity in the hybrid layer detected with in situ zymography. J. Dent. Res..

[51] Liu Y., Tjäderhane L., Breschi L., Mazzoni A., Li N., Mao J., Pashley D.H., Tay F.R. (2011). Limitations in bonding to dentin and experimental strategies to prevent bond degradation. J. Dent. Res..

[52] Breschi L., Maravic T., Cunha S.R., Comba A., Cadenaro M., Tjaderhane L., Pashley D.H., Tay F.R., Mazzoni A. (2018). Dentin bonding systems: From dentin collagen structure to bond preservation and clinical applications. Dent. Mater..

[53] Frankenberger R., Lohbauer U., Roggendorf M.J., Naumann M., Taschner M. (2008). Selective enamel etching reconsidered: Better than etch-and-rinse and self-etch. J. Adhes. Dent..

[54] Feltrin Antoniazzi B., Ferreira Nicoloso G., Larissa Lenzi T., Soares M., Zovico F., de Oliveira Rocha R. (2016). Selective Acid Etching Improves the Bond Strength of Universal Adhesive to Sound and Demineralized Enamel of Primary Teeth. J. Adhes. Dent..

[55] Cuevas-Suárez C.E., da Rosa W.L.O., Lund R.G., da Silva A.F., Piva E. (2019). Bonding Performance of Universal Adhesives: An Updated Systematic Review and Meta-Analysis. J. Adhes. Dent..

[56] Peumans M., Van Meerbeek B., Asscherickx K., Simon S., Abe Y., Lambrechts P., Vanherle G. (2001). Do condensable composites help to achieve better proximal contacts?. Dent. Mater..

[57] Loomans B.A., Opdam N.J., Roeters F.J., Bronkhorst E.M., Burgersdijk R.C., Dorfer C.E. (2006). A randomized clinical trial on proximal contacts of posterior composites. J. Dent..

[58] Loomans B.A., Opdam N.J., Roeters J.F., Bronkhorst E.M., Plasschaert A.J. (2006). Influence of composite resin consistency and placement technique on proximal contact tightness of Class II restorations. J. Adhes. Dent..

[59] Van der Vyver P.J. (2002). Posterior composite resin restorations. Part 3. Matrix systems. S. Afr. Dent. J..

[60] Hancock E.B., Mayo C.V., Schwab R.R., Wirthlin M.R. (1980). Influence of interdental contacts on periodontal status. J. Periodontol..

[61] Ferracane J.L. (2008). Buonocore Lecture. Placing dental composites—A stressful experience. Oper. Dent..

[62] Felix S.A., Gonzalez-Lopez S., Mauricio P.D., Aguilar-Mendoza J.A., Bolanos-Carmona M.V. (2007). Effects of filling techniques on the regional bond strength to lateral walls in Class I cavities. Oper. Dent..

[63] Niu Y., Ma X., Fan M., Zhu S. (2009). Effects of layering techniques on the micro-tensile bond strength to dentin in resin composite restorations. Dent. Mater..

[64] Gonzalez-Lopez S., Lucena-Martin C., de Haro-Gasquet F., Vilchez-Diaz M.A., de Haro-Munoz C. (2004). Influence of different composite restoration techniques on cuspal deflection: An in vitro study. Oper. Dent..

[65] Al Shaafi M., Maawadh A., Al Qahtani M. (2011). Evaluation of Light Intensity Output of QTH and LED Curing Devices in Various Governmental Health Institutions. Oper. Dent..

[66] Rueggeberg F.A. (2011). State-of-the-art: Dental photocuring--a review. Dent. Mater..

[67] Roulet J.F., Price R. (2014). Light curing—Guidelines for practitioners—A consensus statement from the 2014 symposium on light curing in dentistry held at Dalhousie University, Halifax, Canada. J. Adhes. Dent..

[68] Alqabbaa L.M., Alsenani M.S., Alsaif N.S., Alsaif R.A., Binalrimal S.R. (2018). Light intensity output of visible light communication units and clinicians’ knowledge and attitude among Riyadh private clinics. J. Conserv. Dent..

[69] Davidson D.F., Suzuki M. (1999). A prescription for the successful use of heavy filled composites in the posterior dentition. J. Can. Dent. Assoc..

[70] Jackson R.D. (2016). Class II composite resin restorations: Faster, easier, predictable. Br. Dent. J..

