# Supplementary material for: Placement of Posterior Composite Restorations: A Cross-Sectional Study of Dental Practitioners in Al-Kharj, Saudi Arabia
Source: Int J Environ Res Public Health. 2021 Nov 25;18(23):12408. doi: 10.3390/ijerph182312408 (PMC8656557; doi:10.3390/ijerph182312408)
Supplement: Supplementary file 1 [file ijerph-18-12408-s001.zip › ijerph-1444574-supplementary.pdf]

Questionnaire questions:

Q1. Which material do often you use in posterior small cavity (1 or 2 surfaces)?

- Amalgam
- Composite
- Resin modified glass ionomer

Q2. Which material do you often use in posterior large cavity (3 or more surfaces)?

- Amalgam
- Composite
- Other (Indirect restoration)

Q3. Do you often place direct posterior composite restorations in patients with oral para-functional activity?

- Yes
- No

Q4. Do you often place direct posterior composite restorations in patients with poor oral hygiene?

- Yes
- No

Q5. Do you often place direct posterior composite restorations in posterior cavities with 1-2 mm Sub-gingival margins?

- Yes
- No

Q6. Do you prepare a minimum pulpal depth of 2mm for occlusal cavities?

- Yes
- No

Q7. Do you prepare mechanical means of retention for composite restorations?

- Yes
- No

Q8. Do you bevel the occlusal margins of the cavity?

- Yes
- No

Q9. Do you bevel the gingival margin of the cavity?

- Yes
- No

Q10. How often do you achieve the operative field isolation?

- Rubber dam
- Cotton rolls and intraoral suction (Partial isolation)
- Other

Q11. Which adhesive strategy do you use more often?

- Etch-and-rinse (total etch)
- Self-etching (no acid etching)
- Selective enamel etching

Q12. Which placement technique do you often apply for the placement of composite restorations?

- Horizontal layering
- Oblique layering
- Bulk-fill

Q13. Which light-curing unit do you often use to light-cure posterior restorations?

- Quartz tungsten halogen (QTH)
- Light emitting diodes (LED)
- Other

Q14. Do you regularly monitor the output of light-curing unit with a radiometer?

- Yes
- No

Q15. How long do you light-cure composite increment of 2mm thickness?

- 10s
- 15s
- 20s

Q16. For class II composite restorations, after removal of the matrix band, do you often perform additional light-curing from the buccal and lingual directions?

- Yes
- No

Q17. Which matrix system do you often use to restore the proximal contact with composite restoration?

- Sectional matrix
- Tofflemire matrix
- Circumferential matrix
- Other
